# Prevalence, treatment, and neural correlates of apathy in different forms of dementia: a narrative review

**DOI:** 10.1007/s10072-023-07197-7

**Published:** 2023-11-28

**Authors:** Ilaria Parrotta, Stefano Cacciatore, Flavio D’Andrea, Marianna D’Anna, Giulia Giancaterino, Giovanni Lazzaro, Giorgio Arcara, Nicoletta Manzo

**Affiliations:** 1 Movement Control and Neuroplasticity Research Group, Tervuursevest 101, 3001 Louvain, Belgium; 2 IRCCS San Camillo Hospital, Via Alberoni 70, 30126 Venice, Italy; 3 Young Epidemiologists of the Italian Society of Gerontology and Geriatrics (SIGG) (YES) Working Group, Italian Society of Gerontology and Geriatrics, Via Giulio Cesare Vanini 5, 50129 Florence, Italy; 4 Department of Geriatrics, Orthopedics and Rheumatology, Università Cattolica del Sacro Cuore, L.go Francesco Vito 1, 00168 Rome, Italy; 5 Department of Human Neuroscience, Sapienza University, Piazzale Aldo Moro 5, 00185 Rome, Italy

**Keywords:** Alzheimer’s disease, Vascular dementia, Mixed dementia, Parkinson’s disease, Frontotemporal dementia, Behavioral and psychological symptoms of dementia

## Abstract

**Objectives:**

The aim of this review is to provide an overview on prevalence and clinical tools for the diagnosis of apathy, as well as on neurophysiological and neuroimaging findings obtained from studies in patients with apathy in different forms of dementia, including Alzheimer’s disease (AD), vascular (VaD) and mixed dementia, frontotemporal dementia (FTD), and Parkinson’s disease dementia (PDD).

**Methods:**

Randomized controlled trials, non-randomized controlled trials, controlled before–after studies, and interrupted time series from four databases (WebOfScience, Scopus, Pubmed, and PsycINFO) addressing apathy in adults or older people aged over 65 years of age affected by dementia were included.

**Results:**

The prevalence of apathy was 26–82% for AD, 28.6–91.7 for VaD, 29–97.5% in PDD, and 54.8–88.0 in FTD. The assessment of apathy was not consistent in the reviewed studies. Methylphenidate was the most successful pharmacological treatment for apathy. Neurobiological studies highlighted the relationship between both structural and functional brain areas and the presence or severity of apathy.

**Conclusion:**

Apathy is a very common disorder in all types of dementia, although it is often underdiagnosed and undertreated. Further studies are needed to investigate its diagnosis and management. A consensus on the different evaluation scales should be achieved.

## Introduction

Apathy is defined as a decrease in “goal-directed” behavior and impaired motivation not determined by a diminished level of consciousness, cognitive impairment, emotional distress, and depression [1, 2]. Apathy can be a symptom of neurological or psychiatric conditions, although it was recently detected also in older adults with healthy cognitive functions [3], and can display three different phenotypes. “Emotional-affective” apathy is defined as the failure to create the required relationship between emotional-affective impulses and current or future conduct; “cognitive” apathy refers to difficulties in elaborating the plan of actions required for the ongoing or future behavior; “auto-activation” apathy refers to the inability to self-activate thoughts or self-initiate actions, as opposed to a relatively spared ability to generate externally driven behavior [4]. Apathy can be consistently found in several neurodegenerative diseases such as Alzheimer’s disease (AD), Parkinson’s disease dementia (PDD), frontotemporal dementia (FTD), or vascular dementia (VaD) [5]. Its prevalence increases during disease progression [6], and it is linked to faster cognitive deterioration and increased risk of institutionalization [7]. Overall, existing evidence shows that apathy strongly impacts on the quality of life of both patients and caregivers [8]. Nevertheless, data on apathy in different forms of dementia are sparse [9–11]: there is no agreement on a recommended tool for apathy assessment [12, 13], it is rarely investigated as a primary outcome in literature [14–16], its neurobiological correlates are unclear, and evidence on possible treatments is limited [17]. For the above reasons, apathy in dementia is often misdiagnosed and undertreated [18, 19].

The aim of this paper is to provide an overview on prevalence, assessment tools, neurophysiological and neuroimaging correlates, and therapeutic options for apathy in different forms of dementia including AD, PDD, FTD, and VaD. We will identify gaps in the literature and future perspectives and provide information for a proper management of apathy, to improve the quality of life of both patients and caregivers.

## Methods

The following databases were used for this narrative review: WebOfScience, Scopus, Pubmed, and PsycINFO. The script used for the search is (*apathy*) AND (*dementia*). General keywords focusing on the role of apathy in dementia were included.

### Inclusion and exclusion criteria

We included randomized (RCT) and non-randomized clinical trials, controlled before–after studies, and interrupted time series addressing diagnosis, assessment, or treatment of apathy in dementia. Meta-analysis, systematic reviews, and animal models were excluded. Abstracts and full texts from the original search were reviewed individually by different authors. Disagreement at the screening stage was resolved by including a third author at the full-text stage, if required. Studies on subjects with non-neurodegenerative diseases, head trauma, stroke, brain tumors, and psychiatric diseases, as well as those not addressing apathy were excluded. Inclusion criteria entailed studies on adults or older adults (aged over 65 years old) and the presence of cognitive impairment. The research included only articles in English language published after 2017.

### Data extraction

Data from individual studies were extracted in accordance with the PICOS approach [20]. The following information was extracted from each study: (1) author/s and year of publication, type of study, (2) characteristics of the participants, (3) cognitive and/or psychological domains investigated, (4) apathy evaluation, (5) type of treatment or intervention, and (6) primary outcome of the study. Continuous variables are expressed as value ± standard deviation, while categorical variables as absolute number and/or percentage.

## Results

**Fig. 1:**
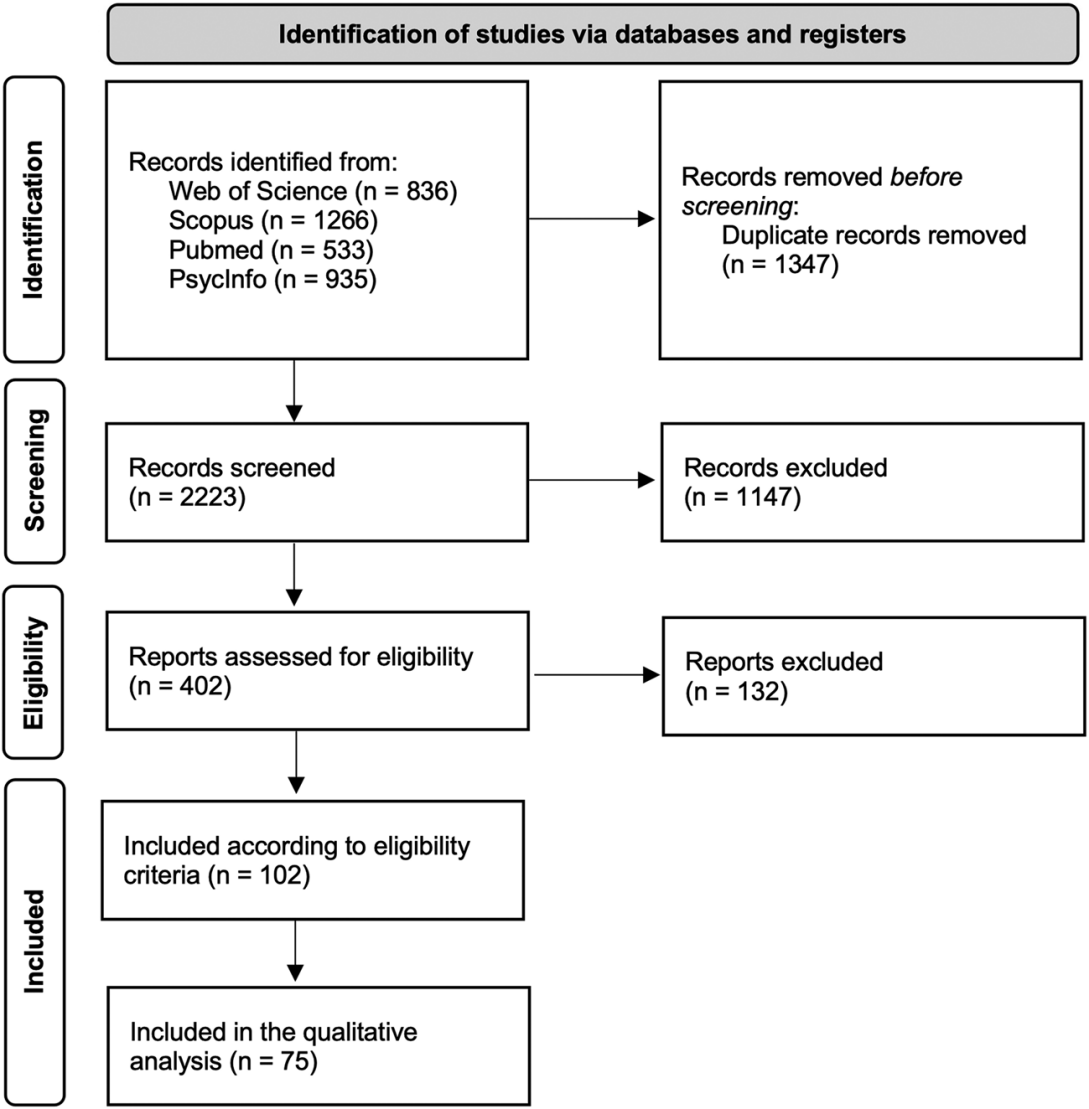
PRISMA flowchart of article selection

The research produced 3570 articles. A total of 836 records were found in Web of Science, 1266 records were identified on Scopus, 533 records were identified through Pubmed, and 935 records were identified on PsycInfo. Identified records were entered into the Mendeley software for their management and elimination of duplicates. A total of 2223 records were screened according to title and abstract. After 1821 records were excluded, 402 articles were evaluated for full-text. Therefore, 102 articles were selected according to eligibility criteria (records excluded 300). Seventy-five articles were included in the qualitative analysis (Fig. 1). We described the main results, according to each major clinical disease. For each clinical condition, after a preliminary description, we reported prevalence, available treatments, tools used for apathy assessment, and results from neuroimaging and functional studies.

### Alzheimer’s disease

AD is a neurodegenerative disease characterized by the accumulation of neuropathological abnormalities such as β-amyloid plaques and neurofibrillary tangles. The prodromal phase of this disease involves neural loss, typically affecting the hippocampus, leading to a progressive atrophy in large-scale of networks widely undermining cognitive and neuropsychiatric functions. AD negatively affects activities of daily living with loss of motivation and interest in several aspects of life. A summary of studies addressing apathy in AD included in the review is shown in Table 1.

**Table 1:** Summary of studies addressing apathy in Alzheimer’s disease and vascular dementia included in the review

Author(s), year	Study design	Dementia	Participants, mean age, male/female (%)	Prevalence, %	Apathy evaluation	Mean apathy score (SD)	Task/other	Primary outcome	Results
Altomari et al., 2022	Cohort study	AD	*n* = 1925 71.58 ± 9 633/1292		NPI	EOAD 12.46 ± 13 LOAD 13.66 ± 13	MMSE ADL	Characterize the presence, overall prevalence, and time of occurrence of BPSD in EOAD versus LOAD;	
Akyol MA et al., 2019	Cross-sectional study	AD FTD VaD	*n* = 46 73.83 (10.28) 26/46 (56.5) *n* = 31 68.13 (9.52) 16/31 (51.6) *n* = 29 74.17 (10.49) 7/29 (24.1)	50.0 66.7 55.2	NPI	4.136 (0.613) 6.227 (0.809) 6.197 (0.787)	CDR MMSE Katz Index of Independence in Activities of Daily Living (ADL) Lawton-Brody Instrumental Activities of Daily Living (IADL)	Compare apathy across AD, FTD, and VaD Determine the factors affecting apathy for AD, FTD, and VaD	Apathy scores in FTD and VaD were significantly higher Apathy scores in AD and VaD positively correlated with cognitive and functional decline No statistically significant relationship between age, gender, and apathy was found
Altunkaya S et al., 2022	Cross-sectional study	SIVD AD	*n* = 23 73.2 (7.55) 10/23 (43.5) *n* = 34 77.8 (6.51) 17/34 (50) Controls (NC) = 23 65.7 (7.42) 11/23 (47.8)		AES AES, Initiation AES, Motivation AES, Socially NPI	SIVD = 45.2 (14.69) AD = 34.4 (13.40) NC = 29.2 (9.34) SIVD = 0.63 (1.29) AD = − 0.08 (0.76) NC = − 0.51 (0.57) SIVD = 0.32 (0.97) AD = − 0.16 (0.93) NC = − 0.07 (1.07) SIVD = 0.09 (1.05) AD = 0.09 (1.01) NC = − 0.22 (0.92) SIVD = 9.3 (8.65) AD = 5.9 (8.23) NC = 4.3 (5.31)	CDR MSE CASI BDI, BAI MRI rs-fMRI	Compare apathy-related functional connectivity (FC) changes among patients with SIVD, AD, and cognitively normal subjects	Significant clinical predictors of apathy were the volume of white matter hyperintensities (WMH), dementia staging, and BDI SIVD patients were more affected in the “initiation” subdomain of the AES Right inferior frontal gyrus, left middle frontal gyrus, and left anterior insula were the critical hubs for apathy Comparison between four different resting state networks (RSNs) showed dissociable FC changes, shared FC in the dorsal attention network, and distinct FC in the salient network across SIVD and AD
Bhat A et al., 2021	Cross-sectional study	VaD LVD SVD	*n* = 76 *n* = 28 56.6 (5.2) 9/28 (32.1%) *n* = 48 58.9 (5.9) 13/48 (27.1%)	LVD = 28.6% SVD = 54.2%	NPI		CDR Kolkata Cognitive Screening Battery FAB MRI	Compare BPSD in LVD and SVD	BPSD were present in 66.67% of patients with SVD and 53.57% of those having LVD Different VaD subtypes have different behavioral profiles Apathy was more common in SVD
Bozgeyik et al., 2018	Cohort study	AD	*n* = 71 78.4 ± 5.8 22/49		NPI	Mean apathy 60.6%	SMMSE GDS NPI ZCBS HAM-D	The aim of the study was to investigate the relationship between behavioral and psychological symptoms of Alzheimer’s disease with caregiver burden and depression	Results show that behavioral and psychological symptoms in Alzheimer’s patients increase the caregiver burden and cause caregiver depression. Preventive measures to prevent the emergence of such symptoms and effective and rapid intervention are required
Jeong et al., 2022	Cross-sectional study	AD	*n* = 59 age 76.5 ± 6.0 years, 17/42	CRD0.5 = 57.6%	NPI	Mean apathy (SD) 2.54 ± 3.54	NPI CDR MMSE MRI SPECT	This study investigated associations between regional cerebral blood flow (rCBF) and neuropsychiatric symptom domains in early AD	The affective domain score was negatively correlated with rCBF in the prefrontal cortex, thalamus, and caudate. The apathy domain score showed inverse correlations with rCBF in the prefrontal and pre/postcentral gyri and midbrain The score of each neuropsychiatric symptom domain showed the differential correlates of brain perfusion, while altered rCBF in the prefrontal cortex was found in all domains
Kazui H et al., 2016	Cross-sectional study	AD DLB VaD FTD	*n* = 1091 76.9 (8.7) 752/1091 (68.9) *n* = 249 78.9 (5.9) 147/249 (59.0) *n* = 156 75.9 (10.0) 76/156 (48.7) *n* = 102 69.9 (8.4) 50/102 (49.0)	CDR 0.5 = 61.2 CDR 1 = 77.8 CDR 2 = 83.5 CDR 3 = 91.1 CDR 0.5 = 62.5 CDR 1 = 79.8 CDR 2 = 82.3 CDR 3 = 100 CDR 0.5 = 52.5 CDR 1 = 87.7 CDR 2 = 90.3 CDR 3 = 91.7 CDR 0.5 = 68.6 CDR 1 = 71.4 CDR 2 = 92.0 CDR 3 = 100.0	NPI	Frequency = 1.9 (1.7) Severity = 1.3 (0.5) ACD = 1.1 (0.9) Frequency = 2.7 (1.7) Severity = 1.5 (0.6) ACD = 1.4 (1.1) Frequency = 3.1 (1.5) Severity = 1.7 (0.7) ACD = 1.9 (1.2) Frequency = 3.6 (1.2) Severity = 2.0 (0.9) ACD = 2.1 (1.3) Frequency = 2.2 (1.8) Severity = 1.5 (0.6) ACD = 1.5 (1.5) Frequency = 2.7 (1.7) Severity = 1.6 (0.6) ACD = 1.6 (1.5) Frequency = 3.2 (1.5) Severity = 2.0 (0.6) ACD = 2.0 (1.3) Frequency = 3.9 (0.5) Severity = 2.0 (0.9) ACD = 2.0 (1.2) Frequency = 2.2 (1.8) Severity = 1.5 (0.6) ACD = 1.5 (1.5) Frequency = 2.7 (1.7) Severity = 1.6 (0.6) ACD = 1.6 (1.5) Frequency = 3.2 (1.5) Severity = 2.0 (0.6) ACD = 2.0 (1.3) Frequency = 3.9 (0.5) Severity = 2.0 (0.9) ACD = 2.0 (1.2) Frequency = 2.3 (1.8) Severity = 1.3 (0.6) ACD = 1.4 (1.5) Frequency = 2.7 (1.8) Severity = 1.5 (0.7) ACD = 1.5 (1.1) Frequency = 3.6 (1.2) Severity = 2.1 (0.8) ACD = 2.0 (1.6) Frequency = 3.9 (0.4) Severity = 2.3 (0.8) ACD = 3.0 (1.2)	CDR NPI-D	Explore the differences of trajectories of 12 BPSD by disease severity in AD, VaD, DLB, and PDD Explore frequency, severity, and associated caregiver distress (ACD) of BPSDs	Trajectories of BPSDs were different in the four major dementias Apathy becomes more severe as dementia progresses in AD, DLB, and VaD Trajectories of BPSDs in FTD were unclear
Manca et al., 2022	Cohort study	AD	*N* = 224 AP-PT *n* = 61 NA-PT *n* = 61 AP-PT 73.33 (6.97) NA-PT 73.93 (8.47) AP-PT 17/44 NA-PT 29/32	CDR AP-PT 0.00 NA-PT 0.50	NPI-Q	AP-PT 5.00 (6) * NA-PT 1.00 (2) *	MMSE NPI CRD	This study investigated the relationship between WM damage and apathy in AD. Sixty-one patients with apathy (AP-PT) and 61 without apathy (NA-PT) were identified from the Alzheimer’s disease	No neurocognitive differences were found between patient groups AP-PT group had more severe neuropsychiatric symptoms. AP-PT had increased WM damage, both macrostructurally, i.e., larger WM hyperintensity volume, and microstructurally, Disruption in structural connectivity might affect crucial functional inter-network communication, resulting in motivational deficits and worse cognitive decline
Manso-Calderón R et al., 2020	Cross-sectional study	AD cVaD sVaD	*n* = 486 79.5 (7.1) 336/486 (69.1) *n* = 136 79.8 (7.2) 62/136 (45.6) *n* = 184 80.3 (6.7) 102/184 (55.4)	Mild = 54.9 Moderate = 66.7 Severe = 59.2 Mild = 55.8 Moderate = 63.5 Severe = 58.6 Mild = 67.4 Moderate = 75.7 Severe = 68.4	NPI		CDR MMSE	Compare BPSD in cVaD, sVaD, and AD	BPSD were present in almost all patients in the subgroups (cVaD 98.5%, sVaD 97.3%, and AD 96.9%), median NPI score of 36 in both cVaD and sVaD and 34 in AD, median number of four BPSD per patient Apathy was the second most frequent disorder (61.8%), after depression (64.4%), and sleep disturbance (60.5%) Higher risk of apathy in cVaD (*p* = 0.007) and sVaD (*p* = 0.0001) than in AD
Palmieri et al., 2023	Clinical trial	AD	*n* = 30 69 9/21		NPI		NPI MMSE CMAI	This clinical trial aimed to enhance the clinical action of THC: CBD cannabis extract administration in AD patients with severe symptoms such as agitation, weight loss, cognitive impairment, and sleep disturbance	The NPI-Q demonstrated a reduction in agitation, apathy, irritability, sleep disturbances, and eating disturbances, consequently improving caregiver distress. Levels of physically and verbally aggressive behaviors, measured using the CMAI questionnaire, were lower in all patients. The MMSSE questionnaire confirmed a significant decrease in cognitive impairment in 45% of the patients
Pillai et al., 2022	Cohort study	AD BPSDs LRP	*n* = 2422 74.4 1446 men (59.7%)		NPI-Q	AD 71.3 LRP 73.1 AD-LRP 75.9	NPI-Q	To determine the clinical phenotypes at the initial visit that are associated with the nature and severity of BPSDs in patients with ADP, LRP, and ADP-LRP	These findings suggest that the risks of BPSDs differ with respect to the initial cognitive phenotype, underlying neuropathology, age, and sex. Awareness of these associations could be helpful in dementia management
Saleh Y et al., 2021	Cross-sectional study	VaD	*n* = 82 68 (11) 37/82 (45)		AES		Addenbrooke’s Cognitive Examination III (ACE-III) BDI Cantril quality of life ladder Effort-based decision-making task MRI DTI analysis	Investigate the association between disruption of effort-based decision-making and apathy Investigate the association between apathy and white matter abnormalities	In apathetic patients, the main criterion driving decision-making was reward magnitude Significant reductions in white matter integrity were related with apathy but not depression Disruption of pathways connecting brain regions implicated in effort-based decision making (“disconnection syndrome”) Both brain and behavioral changes in apathy are associated with drift rate to decision parameter
Santos MAO et al., 2018	Cross-sectional study	VaD MxD	*n* = 53 76.7 (9.4) 19/53 (36)	56.6	NPI		CDR MMSE Cognitive tests battery	Describe the clinical and epidemiological features in a population with VaD and MxD	Apathy was the most frequent NPS (56.6%) Most patients had a single symptom (81.1%), predominantly apathy NPS were more common in mild-to-moderate dementia
Schwertner E et al., 2022	Cross-sectional study	AD (%) VaD (%) MxD (%) DLB (%) PDD (%) FTD (%) Unspecified (UD) (%)	*n* = 3,548 (34.1) 78.3 (8.3) 2443/3548 (68.9) *n* = 1,708 (16.4) 81.5 (7.2) 1015/1708 (59.4) *n* = 1621 (15.6) 81.3 (6.7) 1066/1621 (65.8) *n* = 236 (2.3) 77.6 (6.9) 118/236 (50) *n* = 122 (1.7) 75.9 (7) 47/122 (38.5) *n* = 200 (1.9) 69.8 (9) 111/200 (55.5) *n* = 2970 (28.5) 82.2 (7.1) 1939/2970 (65.3) Total *n* = 10,405 80.2 (7.9) 6739/10,405 (64.8)	1037/3548 (29.2) 521/1708 (30.5) 481/1621 (29.7) 67/236 (28.4) 42/122 (17.8) 77/200 (38.5) 895/2970 (30.1) 3120/10,405 (30)	NPI No symptom (0) Mild symptoms (1–3) Clinically significant (> 3)	No = 2434 (70.1) Mild = 374 (10.8) C.S. = 663 (19.1) No = 1157 (69) Mild = 190 (11.3) C.S. = 331 (19.7) No = 1110 (69.8) Mild = 205 (12.9) C.S. = 276 (17.3) No = 165 (71.1) Mild = 32 (13.8) C.S. = 35 (15.1) No = 79 (65.3) Mild = 24 (19.8) C.S. = 18 (14.9) No = 119 (60.7) Mild = 22 (11.2) C.S. = 55 (28.1) No = 2028 (69.4) Mild = 372 (12.7) C.S. = 523 (17.9) No = 7092 (69.4) Mild = 1219 (11.9) C.S. = 1901 (18.6)	MMSE Medications	Characterize BPSD in AD, VaD, MD, PDD, DLB, FTD, and UD in individuals residing in long-term care facilities	VaD and FTD subgroups had a higher risk of apathy compared to AD
Tay J et al., 2019	Cross-sectional study	VaD	*n* = 331 68.9 (8.3) 194/331 (58.6)		AES	27.3 (7.8)	MMSE Center for Epidemiologic Studies Depression Scale (CESD) MRI	To investigate whether white matter network disruption underlies the pathogenesis of apathy, but not depression, in sVaD	Disruption of pathways connecting brain regions implicated in effort-based decision making (“disconnection syndrome”) Impaired connectivity in premotor and cingulate regions Apathy, but not depression, is associated with white matter tract disconnection in SVD
Tu MC et al., 2017	Cross-sectional study	sVaD AD	*n* = 24 71.71 (12.01) 11/24 (45.8) *n* = 32 73.59 (8.00) 17/32 (53.1)		NPI	Apathy symptom All = 0.65 (0.89) CDR 0.5 = 0.25 (0.80) CDR 1–2 = 1.20 (0.72) Apathy domain All = 0.96 (1.43) CDR 0.5 = 0.07 (0.27) CDR 1–2 = 2.20 (1.48) Apathy symptom All = 0.23 (0.51) CDR 0.5 = 0.21 (0.46) CDR 1–2 = 0.27 (0.61) Apathy domain All = 0.25 (0.62) CDR 0.5 = 0.29 (0.72) CDR 1–2 = 0.18 (0.41)	CDR MMSE CASI MRI DTI analysis	Compare BPSD between patients with sVaD and Alzheimer’s disease (AD) across stages Explore the associations with DTI in the corpus callosum (CC) and other major fibers	Disruption of pathways connecting brain regions implicated in effort-based decision making (“disconnection syndrome”) Apathy severity positively correlated with dementia severity and global cognition decline Apathy was more severe in sVaD than AD Disruption of right superior longitudinal fasciculus predicted the apathy domain

*AD* Alzheimer’s disease, *AES* apathy evaluation scale, *BAI* Beck Anxiety Inventory, *BDI* Beck’s Depression Inventory, *BPSD* Behavioral and Psychological Symptoms of Dementia, *CASI* Cognitive Abilities Screening Instrument, *CDR* Clinical Dementia Rating scale, *cVaD* large-vessel or cortical vascular dementia, *DLB* dementia with Lewy bodies, D*TI* Diffusion Tensor Images, *EOAD* Early Onset Alzheimer Disease, *FAB* Frontal Assessment Battery, *FTD* frontotemporal dementia, *LOAD* Late Onset Alzheimer Disease, *LRP* Lewy body-related pathology, *LVD* large vessel disease, *MMSE* Mini Mental State Examination, *MRI* magnetic resonance (structural), *MxD* mixed dementia, *NPI* neuropsychiatric inventory, *NPI-D* NPI caregiver distress, *PDD* Parkinson’s disease dementia, *rs-fMRI* resting state functional MRI, *SIVD* subcortical ischemic vascular disease, *sVaD* small-vessel or subcortical vascular dementia, *SVD* small vessel disease, *VaD* vascular dementia

#### AD—prevalence

Thirty-nine articles met the inclusion criteria for apathy in AD. Five of them investigated AD together with bvFTD [12, 21–24], primary progressive aphasia (PPA) [23], and subcortical ischemic vascular disease (SIVD) [13]. The prevalence of apathy varied from 26 to 82%, and it increases, along with its severity, with the progression of cognitive and functional decline and brain atrophy.

#### AD—apathy assessment

Seven apathy-specific scales were used: Apathy Evaluation Scale (AES) [13, 23, 25–31], Dementia Apathy Interview and Rating (DAIR) [32, 33], Dimensional Apathy Scale (DAS) [22, 34, 35], Lille Apathy Rating Scale (LARS) [12], Apathy Rating Scale (ARS) [36], Apathy in Dementia-Nursing Home Version Scale (AES-NH) [37]. Seven apathy subscales were included in more global questionnaires, such as Neuropsychiatric Inventory (NPI) [12, 21, 24, 32, 33, 38–50] or Frontal Behavioral Inventory (FBI-a) [12]. The most utilized tools were AES and NPI. Using AES, the highest score was reported in a sample of 32 patients (mean age 84.5 ± 9.5 years old) and was 56.5 ± 13.0 points [31], while the lowest score of 34.4 ± 13.40 points was found in a sample of 34 patients (with a lower mean age of 77.8 ± 6.51 years old) [13]. The highest NPI score was 7.8 ± 2.4, and it was observed in a sample of 200 patients aged 76 ± 5 years old [45], and the lowest NPI score was 0.6 ± 0.8, and it was reported in a sample of 95 subjects aged 75.6 ± 7.4 years [39]. In a large sample of 1925 patients suffering from AD, apathy was diagnosed in more than two third of the sample [47].

#### AD—apathy treatment

Nine studies addressed different therapeutic options, and methylphenidate was the most investigated treatment.

##### Methylphenidate

Efficacy of methylphenidate was investigated by two RCTs. Padala and colleagues demonstrated an increase on the clinician/researcher version of the AES after 12 weeks of treatment with 10 mg/day of methylphenidate. A greater improvement in apathy in multiple domains (behavioral, cognitive, emotional, and motivation), global cognition, functional status, caregiver burden, and depression started after 4 weeks [51]. Mintzer et al. in a 6-month RCT demonstrated significant beneficial effects of 20 mg/day methylphenidate on apathy. The largest decrease in the NPI apathy score was observed during the first 100 days. Conversely, no further improvements in cognitive measures and quality of life was reported after 6 months [45].

##### Sertaline

An RCT investigated the efficacy of sertraline, escitalopram, and nicergoline to treat apathy and depression in AD. Regarding apathy, the authors observed a significant improvement with sertraline only [52].

##### Ninjin’yoeito (NYT, TJ-108)

An open-label pilot study reported significant positive effects on apathy after using Ninjin’yoeito (NYT, TJ-108) in 20 AD subjects. NYT, TJ-108 is a traditional Japanese multicomponent herbal medicine. The authors reported an increase on NPI apathy and anorexia subscales and global cognitive functions [38].

##### BrainUp-10® (BU-10)

An RCT by Guzman-Martinez and colleagues investigated the effect of BrainUp-10® (BU-10) in 74 AD subjects. After 24 weeks of treatment, the authors obtained a statistically significant improvement of apathy. In contrast, no differences on cognitive functions were observed [53].

##### Oil diluted cannabis extract (Bedrocan)

Palmieri and Vadalà demonstrated benefits on apathy-subdomain score of NPI and cognitive status assessed by mini-mental state examination (MMSE) (in 45% of the sample), as well as on the caregiver’s quality of life, 3 months after the administration of Bedrocan [49].

##### Stimulation techniques

Two studies investigated the effect of repetitive transcranial magnetic stimulation (rTMS), showing significant effects on apathy [30, 40]. Nguyen and colleagues examined the efficacy of 5 weeks of rTMS combined with cognitive training in a sample of 10 AD patients. rTMS was delivered over 6 brain areas, namely, right and left dorsolateral pre-frontal cortex (DLPFC), right and left posterior parietal cortex associative areas, and Broca and Wernicke language areas, and it was combined with cognitive tasks stimulating the three different cortical regions (spatial attention tasks for the parietal cortex, naming of actions and objects, word recall and spatial memory tasks for the prefrontal cortex, and syntax and grammar tasks for language areas). For each region, 40 s of cognitive training were performed between each train of 10 Hz-rTMS stimulation. A series of 20 trains per session was administered, and the patients received 3 sessions. At the end of the treatment, a significant improvement of apathy, disability, and cognitive functions was observed [40]. A study on 20 AD patients by Padala and colleagues reported significantly greater improvement of apathy and general cognitive functions after 4 weeks of rTMS (10 Hz on DLPFC). Positive effects started after 4 weeks of rTMS treatment and were still durable at 12 weeks of follow-up [30].

##### Other treatments

Inel Manav and colleagues evaluated the effect of reminiscence therapy on apathy and cognitive performance in a cohort of 72 mild AD subjects. The protocol involved internet-based videos for 60 min once a week for 3 months and resulted in improving both apathy levels and cognitive functioning [36]. A pilot study showed the positive effects of 10 weeks of horticultural therapy on apathy, cognitive, and functional abilities in 32 AD subjects. Over the course of 10 weeks of activities, a statistically significant reduction in apathy was observed in the experimental group [31].

#### AD—neurological correlates of apathy

Four magnetic resonance imaging (MRI) studies identified the brain regions associated with apathy in subjects with AD. In patients with moderate levels of apathy, Huey and colleagues found a greater degree of atrophy in ventromedial and ventrolateral prefrontal cortex (PFC), posterior cingulate cortex (PCC), and the superior temporal sulcus. These regions entailed brain networks responsible for arousal, threat response, and reward processing [24]. Differently, a study in patients with moderate to severe AD and with moderate apathy, performed using tensor imaging (DTI), showed bilateral damage associated with the severity of apathy in the corpus callosum and internal capsule [37]. Wei and colleagues found specific grey matter (GM) atrophy for each apathy subdomain. Emotional apathy involved cerebellum, ventral PFC, and the amygdala; executive apathy entailed left orbito-frontal cortex (OFC), bilateral frontal pole, lateral temporal regions including the right middle temporal gyrus and temporal pole, as well as the left supramarginal and angular gyri; initiation apathy involved right medial PFC, left frontal pole, OFC, the right paracingulate, and anterior cingulate cortex (ACC) [22]. Kumfor and colleagues identified three different apathy domains through NPI, Cambridge Behavioral Inventory (CBI-R), and Disability and Dementia scale (DAD). For affective apathy, GM atrophy was detected in the left temporal poles, extending into the bilateral OFC, subcallosal cortex, and bilateral insula; behavioral apathy in the frontal cortex and subcortical areas, including the left caudate, extending into the nucleus accumbens, the right precentral gyrus, and the cerebellum; cognitive apathy in left orbitofrontal and subcallosal regions, extending dorsally into the medial PFC, anterior cingulate and superior frontal gyrus, inferior temporal gyrus, and posterior cingulate cortex [21].

Three studies used resting state functional MRI (rs-fMRI) to evaluate cerebral functional connectivity (FC) underlying apathy in AD. FC is a measure of how different brain regions interact with each other [54]. Buyukgok and colleagues compared 10 apathy-early-stage-AD subjects to 10 non-apathy-AD subjects and 10 cognitively normal groups and addressed a significant hypo-functioning at a trend-level in the anterior component of default mode network (DMN), specifically in the preangular ACC in apathy-AD subjects [44]. In contrast, another rs-fMRI study on subjects with mild to severe AD did not detect any decrease in the activity of DMN between subjects with apathy (*n* = 35) and subject without apathy (*n* = 35). Downregulation of DMN, which is a typical feature of AD, was observed only between AD and the healthy control group. Moreover, reduced connectivity between the left insula and the right superior parietal cortex and increased connectivity between DLPFC and the right superior parietal cortex emerged [41]. The different findings of the two studies may be due to several reasons. First, different characteristics of the sample and small sample size; second, different methods used to detect the networks; third, taking into account that the down-regulation is seen only at a trend-level, it may be possible that DMN does not mediate apathy in AD as hypothesized by Buyukgok and colleagues [44]. A recent study of Altunkaya and colleagues described the right inferior frontal gyrus (FG), left middle FG, and left anterior insula as critical hubs for apathy in AD [13].

Yeh and colleagues used proton magnetic resonance spectroscopy to investigate apathy-related neurochemical alterations. They explored only the frontal brain regions and discovered that each subdomain of apathy was associated with neurochemical variations in the ACC, without alterations in the OFC [26].

Jeong et al. assessed the association between regional cellular blood flow (rCBF) and neuropsychological symptoms (NPS) in early AD using single-photon emission computed tomography (SPECT) in a sample of 59 patients. Results showed apathy was associated with a decrease rCBF in prefrontal, pre/postcentral, and midbrain areas [48]. Apathy-related rCBF reduction in the midbrain may be one of the novel findings in patients with early AD. This finding supports previous evidence from preclinical and clinical studies suggesting that alterations in the midbrain structure and the dysfunction of the dopaminergic system may result in the expression of apathy.

Three studies using positron emission tomography (PET) in apathy-AD subjects showed dysfunction of ACC [12, 28], DLPFC [28], and OFC [55]. More specifically, Fernández-Matarrubia and colleagues compared the features of apathy in patients with behavioral variant of FTD (bvFTD) and AD [12]. Their study demonstrated that patients with bvFTD displayed lower metabolism in the left lateral prefrontal cortex, medial frontal/anterior cingulate, and OFC and anterior insular cortices, while patients with AD were characterized by dysfunction of medial/anterior cingulate circuit without an involvement of OFC and DLPFC [12]. According to the authors, those differences may be accountable for the greater impairment of the emotional dimension observed in apathy in bvFTD compared with AD patients [12]. On the other hand, Marshall and colleagues found an association between apathy and small tau clusters within the right ACC and DLPFC, which were more pronounced in individuals with greater amyloid burden [28]. Finally, according to Kitamura and colleagues, tau tangle formation in OFC was found in patients with apathy in AD. For this reason, the authors suggest a possible therapeutic role of novel antitau drugs on apathy; however, further neuroimaging studies are needed to monitor structural and functional changes in neuronal regions involved in apathy with and without therapeutic interventions [55].

### Vascular and mixed dementia

VaD is the second most common dementia worldwide after AD, accounting for 15–20% of all cases in North America and Europe [56, 57]. Recent studies identified a continuum between VaD and AD, so that the coexistence of the two is defined as mixed dementia (MxD). Although pure AD and pure VaD can be diagnosed with good accuracy, identifying mixed forms can be challenging [58, 59]. In this review, we included 10 studies about VaD and MxD. Among them, only two analyzed the MxD subgroup [10, 60] (Table 1).

#### VaD—apathy prevalence

Akyol et al. estimated a prevalence of 55.2% [61]. Similarly, Santos and colleagues addressed a prevalence of 56.6% [60]. A large study by Schwertner and colleagues on 10,405 patients with dementia estimated a prevalence of 30.5% among individuals affected by VaD (*n* = 1.708) and 29.7% in MxD (*n* = 1.621) [10]. Two studies reported apathy prevalence according to VaD subtype: Bhat et al. compared large vessel disease (LVD) versus small vessel disease (SVD) (28.6%vs 54.2%) [62], while according to Manso-Calderon et al., apathy was more frequent in subcortical VaD (sVaD) than in cortical VaD (cVaD) [63]. Kazui et al. stratified the prevalence of apathy according to dementia severity, assessed through the Clinical Dementia Rating scale (CDR). For VaD, they reported 52.5% for CDR 0.5, 87.7% for CDR 1, 90.3% for CDR 2, and 91.7% for CDR 3 [64].

#### VaD—apathy assessment

The most used tool for apathy evaluation was NPI (*n* = 8) [10, 13, 60–65]. Three studies used the AES [13, 66, 67]. One study used both NPI and AES [13]. Tu and colleagues stratified NPI scores according to CDR in SIVD, reporting higher scores for both the symptom and the domain of apathy in more severe dementia [65]. According to Schwertner and colleagues, of the 1708 patients affected by VaD, 11.3% suffered from mild apathy (NPI 1–3), while for 19.7% apathy was clinically significant (NPI > 3). Among 1621 individuals with MxD, 12.9% suffered from mild apathy, while for patients 17.3% apathy was clinically significant [10]. Altunkaya and colleagues analyzed the prevalence of apathy subdomains, obtained by grouping the 18 items composing AES into “initiation” (factors involved in the act of beginning an action), “motivation” (the reason for acting), and “socially” (social involvement). They reported a mean total score of 45.2 ± 14.69 and a score of 0.63 ± 1.29 for “initiation,” 0.32 ± 0.97 for “motivation,” and 0.09 ± 1.05 for “socially” [13]. To analyze the behavioral pattern in apathy and compare it to depression, Saleh et al. designed an effort-based decision-making task via psych-toolbox (psychtoolbox.org) [66].

#### VaD—apathy treatments

No studies explored apathy treatments as primary outcomes in VaD.

#### VaD—neurological correlates of apathy

Seven studies used MRI to evaluate ischemic brain damage and structural and functional variations. Tu and colleagues and Saleh and colleagues analyzed DTI parameters like fractional anisotropy (FA) and mean diffusivity (MD) [65, 66]. Altunkaya et al. performed brain mapping using rs-fMRI to evaluate FC between four different resting state networks (RSNs), which are areas of the brain showing synchronous activity at rs-fMRI, in patients with SIVD and AD [13]. Akyol et al. investigated the relation between apathy and basic and instrumental activities of daily living. According to their study, cognitive and functional decline were risk factors associated with apathy, while no statistically significant relationship was found between age, gender, and apathy [61]. According to Altunkaya et al., the volume of white matter (WM) hyperintensities (WMH), dementia staging, and Beck Depression Inventory (BDI) were significant clinical predictors of apathy [13]. Saleh et al. found that in patients with apathy, the main criterion driving decision-making was reward magnitude, as they were less responsive to low rewards and high efforts. In apathy, there was a reduction of drift rate to the decision parameter (the rate of evidence accumulation), and this change positively correlated with WM alterations. This means that patients with apathy spent less time accumulating evidence before taking a decision, and action was rejected in a shorter time, as the reward was less appealing [66]. Tu et al. found that disruption of the right superior longitudinal fasciculus predicted apathy [65]. Tay et al. showed an impaired connectivity in premotor and cingulate regions [67].

### Parkinson’s disease

Parkinson’s disease (PD) is caused by degeneration of dopaminergic neurons of the substantia nigra and striatum and manifests with cardinal motor symptoms (bradykinesia, rigidity, tremor, gait/postural instability), as well as non-motor symptoms, including NPSs and autonomic dysfunction [68]. Apathy, together with depression, is one of the most common NPSs both in PD and PDD [15, 16]. We found ten studies addressing apathy in PDD. All of them addressed apathy and common NPSs in PDD, and none of them exclusively investigated apathy. A summary of studies addressing apathy in PDD included in the review is shown in Table 2.

**Table 2:** Summary of studies addressing apathy in Parkinson’s disease dementia included in the review

Authors, publication year	Study design	Dementia	Participants, mean age (SD), female (%)	Prevalence of apathy, %	Apathy evaluation	Mean apathy score (SD)	Task/other	Primary outcome	Results
R. Moretti et al. 2017	RCT	PDD	*n* = 48 70.4 (2.34) 20/48 (14.6)	*T*_0_ = 77% *T*_1_ = 62% *T*_2_ = 69%	AES-C AES-S NPI	AES-S: *T*_0_ = + 16.3 (4.1) *T*_2_ = + 19.9 (2.1) AES-C: *T*_0_ = + 15.5 (3.7) *T*_2_ = + 21.5 (2.7) NPI T_0_ Frequency x severity = 8 Caregiver distress = 4 T_2_ Frequency × severity = 8 Caregiver distress = 8 NPI apathy score AES-S *r*: 0.71 *p* < 0.01 NPI apathy/ AES-C *r*: 0.78 *p* < 0.01 FAB/apathy (NPI + AES-C) *r*: 0.75 *p* < 0.01 For NPI *r*: 0.75 *p* < 0.01 For AES-C *r*: 0.79 *p* < 0.01	FAB MoCA NPI LEDD	Evaluate effect of Rivastigmine for improving BPSD and apathy in PDD	Apathy as most relevant symptom in PDD Sever impact of Apathy on daily living and severe revelance for caregivers Rivistagmine reduces apathy scores in PDD at 6moth but apathy scores increase at 12 months Rivastigmine does not ameliorate score of apathy but reduces other NPS signs (irritability, disinhibition, and euphoria)
C.H.F Camargo et al. 2017	Cross-sectional study	PDD	*n* = 49 69.55 (11.37) 8/49 (43.5) *n* = 34 77.8 (6.51) 17/34 (50) Controls (NC) = 23 65.7 (7.42) 11/23 (47.8)	Apathy and NoDepression: 30% MildDepression: 35% ModerateDepression: 27.5% SevereDepressiom: 5% Total: 97.5%	AES	PDD SOPA-COG (< 22) :21.43 (3.85) AES vs SCOPA-COG *r* = − .2578 AES vs UPDRS-III *r* = − .4390 AES and motor changes and cognitive impairment: *r* = − .4089	UPDRS-III SCOPA-Cogn MADRS	Identify the characteristics of depression and apathy in patient with and without PDD	Apathy in PDD is more prevalent than depression Apathy has a greater association with more advanced dementia Apathy can be present with or without depression
R. Modreanu et al. 2017	Cohort study	PDD	PD *n* = 58 Not shown 28(48%) PDND at baseline: *n* = 37 Not shown 14 (38%) PDD at baseline: *n* = 21 Not shown 14(67%)	At baseline: Moderate to severe apathy: PDND = 11% PDD = 28% 18-month follow-up: Absent-to-mild MFs = 20.5% Moderate-to-severe MFs = 11.8%	UPDRS	Not presented	CSF LEDD MFs UKPDS	Identify the correlation between MFs, PD phenotype, CSF markers (τ, Aβ), and dementia risk in PD	No significant correlation between levels of apathy and CSF markers Levels of apathy do not predict progression to dementia
J. Wojtala et al. 2018	Cohort study	PDD	*n* = 538 67.9 ± 7.0 173 (32.2) PD–N: -TR-D *n* = 24 67(6.9) 50.0% -PIGD-D *n* = 183 66.2(7.3) 31.7% -ND *n* = 15 65.5(9.4) 53.3% PD-MCI: -TR-D *n* = 15 66.4(6.8) 33.3% -PIGD-D *n* = 202 68.6(6.4) 28.7% -ND *n* = 26 66.3(6.9) 34.6% PD-D: -PIGD-D *n* = 70 72.2(5.2) 32.9% -TR-D *n* = 1 -ND *n* = 1	Not shown	AES	PD–N: 28.2(7.5) -PIGD-D 28.1(7.2) -TR-D 26.3(7.2) -ND 31.9(10) PD-MCI: 31.6(9.9) -PIGD-D 31.5(9.7) -TR-D 32.1(9.8) -ND 31.8(11.7) PD-D: 38(10.3) -PIGD-D 38.3(10.5) -TR-D 29 -ND 36(1.4)	MMSE UPDRS (parts I, III, IV) EQ-5D GDS PDQ-39 LEDD MMSE PANDA CERAD (Word list learning, delayed recall and recognition, delayed recall of copied figures, phonemic and semantic verbal fluency, figure copying) Stroop interference task BTA WMS-R forward and backward TMT-A TMT-B TMT- B/A MCST LPS 50 BNT MCST	Examine cognitive profiles among patients with PD by motor phenotypes and its relation to cognitive function	Apathy scores do not differ between three PD phenotypes
R.P Barbosa et al. 2019	Cross-sectional study	PDD	PD *n* = 128 73.20(8.70) 65/128 (50.78) PDD *n* = 21 77.90(5.97) 61.9% PD-MCI *n* = 31 74.26(7.03) 51.6% PDCN *n* = 76 71.29(9.45) 47.4%	Not shown	AS	PDCN: 10.74(6.48), *p* < 0.001 PDMCI: 12.10(8.74), *p* < 0.001 PDD: 15.89(9.73), *p* < 0.001 PDCNC vs. MCI *p* = 0.833 PDCN vs. PDDD *p* < 0.001 PDMCI vs. D *p* = 0.099 PDCN with SCC 11.62(6.39), *p* = 0.007 PDCN without SCC 6.46(5.27), *p* = 0.007 PDMCI with SCC 11.8(9.18), *p* = 0.448 PDMCI without SCC 13.20(6.69), *p* = 0.448	UPDRS II and III MoCA ADL HADS-A HADS-D NMSS LEDD H&Y	Determine the prevalence of SCC in a PD and investigate the association between SCC and cognitive impairment in PD Asses the associated factors with SCC in non demented PD	PDD and PDMCI do not differ in SCC In PDCN, the presence of SCC is associated with lower MoCA scores and apathy scores SCC severity associates with depression (*r* = 0.365, *p* = 0.001), apathy (*r* = 0.423, *p* < 0.001), and anxiety severity but not with MoCA score
M.C Campbell et al.2020	Cohort study	PDD	*n* = 162 66.1 (7.7) 38,3% Motor only *n* = 63 63.2 (6.3) 52% Psychiatric and motor *n* = 17 64.5 (8.8) 23% Cognitive and motor *n* = 82 68.6 (7.6) 30%	Not shown	FrSBe-A NPIQ	Not shown	LEDD CDR MMSE BSI UPDRS-3 Digit Span Digit Symbol CVLT-II short form Logical Memory BTN Judgment of Line Orientation Spatial Relations Test TMT Verbal Fluency-Switching Color-Word Interference GDS	Determine the key features that best discriminate PD subtypes using Latent class analysis (LCA) Analyze the utility of PD subtypes to predict clinical milestones	Depression, executive function, apathy, bradykinesia, visuospatial, attention and PIGD best discriminated subtypes with 95.1% correct classification Depression, executive function, and apathy, classification accuracy is 89.5% Depression and apathy increase mortality risk in Parkinson’s disease
P. Bugalho et al.2021	Cohort study	PDD	*n* = 72 70.22(9.09) 46.6%		Apathy scale	*T*_0_ = 12.13 (8.29) *T*_1_ = 16.25 (10.13) *p* = 0.004 %0.25 e.s. = − 0.50 AS and: MoCA = − .009 (− 0.154 to 0.13) *p* = 0.902 Cognitive state = 1.011 (0.936 to 1.092) *p* = 0.787 Disability = 0.052 (− 0.538 to 0.643) *p* = 0.860 HRQL = .008 (0.000 to 0.016) *p* = 0.042 Motor function = − 0.025 (− 0.089 to 0.139) *p* = 0.661	DED NMSS UPDRS-II and III MoCA SCOPA- Sleep RSBD-Q HADS PPQ H&Y EQ – EuroQol	Investigate the evolution of motor and non-motor symptoms in PD patients Identify the predictors of motor, cognitive, disability and health-related quality of life Estimate the utility of a battery of separate NMS scales (BSS) versus the Non-Motor Symptom Scale (NMSS)	After four years there is a significant worsening of apathy (medium effect size) Disease duration is significantly related with symptom progression regarding RBDSQ scores and UPDRS II scores
Horne et al. 2021	Cohort study	PDD	*n* = 328 PD–N *n* = 180 68(8) 63/117 PD-MCI *n* = 108 70(7) 82/108 PDD *n* = 40 74(7) 30/40	Not shown	NPI	PD–N = 0.7 (1.8) PD-MCI = 0.7(1.7) PDD = 2.1 (2.9)	LEDD ADAS-Cog CDR DRS-2 (AESS) GDS IADL MoCA PDQ UPDRS WTAR UPDRS	Study the association of neuropsychiatric symptoms in PD patients and the conversion to PDD	PDD group has more severe apathy to PD–N Similar overall pattern between PDD and PD-MCI Apathy scores do not differ significantly among PD–N and PD-MCI There is no significant association between apathy measures and the progression to PDD
C.S. Severiano E Sousa et al. 2021	Cross-sectional study	PDD	*n* = 85 75.4 (6.9) 49/85 (57.6%)	51.8%	NPI	3.6 (3.6)	H&Y S&E GDS DSM-IV DSM-5 MMSE Pill Questionnaire ADL Digit span Similarities (WAIS-III) Phonologic fluency TMT (A and B) FAB RAVLT BNT Copy of the clock Benton line orientation test Benton face recognition test	Evaluate the frequency of dementia in late-stage PD patients Assess the impact of using different diagnostic criteria	High discrepancy on the frequency of dementia depending on the criteria applied
Y. Tajiri et al. 2021	Case–control study	PDD	*n* = 49 Not shown 25/49 Non-progressive PD-MCI: *n* = 33 Not shown 18/33 Progressive PD-MCI: *n* = 33 Not shown 7/16	Not shown	Apathy scale	Overall: 15.73 (11.50–19.00) Non-progressive PD-MCI: 15.33 (11.50–19.50) Progressive PD-MCI: 16.56 (11.00–19.00) *p* = 0.528	MMSE MoCA-J UPDRS-III GDS PSQI ESS RBDSQ-J	Identify the predictors of progression from PD-MCI to PDD	Apathy scores do not predict progression from PD-MCI to PDD

*ADAS-Cog* Alzheimer’s Dementia Assessment Scale-Cognitive, A*DL* Activities of daily living, *AES* apathy evaluation scale, *AES-C* Clinician/Researcher Rated Version of the Apathy Evaluation Scale, *AES-S* self-report version of apathy), *AS* Apathy Scale, *BAI* Beck Anxiety Inventory, *DRS-2* (*AESS*) Dementia Rating Scale-2 (Age and Education Scaled Score), *BNT* Boston Naming Test, *BTA* Brief Test of Attention, *BDI* Beck’s Depression Inventory, *BPSD* Behavioral and Psychological Symptoms of Dementia, *CERAD* +  Consortium to Establish a Registry for Alzheimer’s Disease-PlusLPS 50 + , *CSF* cerebrospinal fluid, *CVLT-II* California Verbal Learning Test-II, *CASI* Cognitive Abilities Screening Instrument, *CDR* Clinical Dementia Rating scale, *DED* L-dopa equivalent daily doses, *FAB* Frontal Assessment Battery, *FrSBe-A* Frontal Systems Behavior Scale – Apathy Subscale, *HADS* Hospital Anxiety and Depression Scale, *DSM-5; ESS* Epworth Sleepiness Scale, *GDS* Geriatric Depression Scale, *LEDD* levodopa equivalent daily dose, LPS 50 + , Leistungsprüfsystem for aged 50 + (subtest 7: spatial rotation, subtest 9: spatial imagination), *H&Y*, Hoehn and Yahr scale, *LVD* large vessel disease, *MADRS* Montgomery-Åsberg Depression Rating Scale, *MCST* Modified Card Sorting Test, *MFs* Lesser motor fluctuations, *MMSE* Mini Mental State Examination, *MoCA* Montreal Cognitive Assessment, *ND* not determined, *NMSS* Non-Motor Symptom assessment Scale for Parkinson’s disease, *NPI* neuropsychiatric inventory, *NPI-D* NPI caregiver distress, *NPIQ* Neuropsychiatric Inventory Questionnaire, *PANDA* Parkinson Neuropsychometric Dementia Assessment, *PIGD-D* postural instability and gait difficulty, *PD* Parkinson’s Disease, *PDCN* PD-cognitively normal, *PDMCI* PD-mild cognitive impairment, *PDD* PD-dementia, *PDQ* Parkinson’s disease Questionnaire, *PPQ* Parkinson’s Psychosis Questionnaire, *PSQI* Pittsburgh Sleep Quality Index, *RAVLT* Rey Auditory and Verbal Learning Test, *RBDQ-J* rapid eye movement sleep behavior disorder screening questionnaire [Japanese version], *SCC* Subjective cognitive complaints, *SCOPA-Cogn*: Scales for Outcomes in Parkinson’s Disease-Cognition, *TMT* Trial Making Test, *TMT-A* Trail Making Test version A, *TMT-B* Trail Making Test version B, TMT-B/A, *TR-D* tremor-dominant, *UPDRS* Unifed Parkinson’s Disease Rating Scale, *UPDRS-3* Unifed Parkinson’s Disease Rating Scale-3, *UPDRS-II* Unifed Parkinson’s Disease Rating Scale II, *UPDRS-III* Unifed Parkinson’s Disease Rating Scale III, *UKPDS* United Kingdom Parkinson’s Disease Society, *WAIS-III* Wechsler Adult Intelligence Scale 3rd Edition, *WTAR* Wechsler Test of Adult Reading for premorbid IQ, *WMS-R* Wechsler Memory Scale-Revise

#### PD and PDD—apathy prevalence

According to Modreanu and colleagues, the prevalence of moderate-to-severe apathy was 28–40% in PD and 29–52% in PDD [11]. Two studies reported a percentage of 77% [16] and 97.5% [15] in PDD.

#### PD and PDD—apathy assessment

Three studies used NPI [16, 69, 70]. Nine studies used apathy-specific screening tools, and among them, the Starkstein’s Apathy Scale (AS) was the most frequently used [11, 68, 71, 72]. Two studies used the AES [15, 73]. One study used the Frontal Systems Behavior Scale—Apathy subscale [70]. Studies using NPI showed lower prevalence than those using apathy-specific scales. On the other hand, Moretti and colleagues used both AES clinician and caregiver versions and addressed the highest rates of apathy [16]. Campbell et al. used a general screening tool for psychiatric functions and an apathy-specific tool; however, the authors did not report the scores [70]. Bugalho and colleagues reported a significant worsening of apathy during a follow-up period of 4 years [72], while Horne et al. did not observe significant differences during the same interval [69].

According to three studies, in patients with PDD, apathy was more severe than in subjects with PD-mild cognitive impairment (PD-MCI) and cognitively normal PD patients (PD-CN) [11, 68, 73]. In contrast, Barbosa and colleagues found similar scores among PD-CN, PD-MCI, and PDD [71], and Camargo and colleagues did not observe differences in severity in PD subjects with and without dementia [15]. Interestingly, Barbosa and colleagues found different scores among participants with and without subjective cognitive impairment (SCI). Additionally, both PD and PD-MCI subjects with SCI reported higher apathy scores than same-category participants not addressing SCI [71]. No association was found between baseline apathy measures and future dementia risk [15, 69], as well as between demographic characteristics (such as age and gender) and levels of apathy. Patients progressing to dementia had significantly greater proportions of moderate-to-severe depression, visual hallucinations, memory complaints, and non-motor predominance at baseline compared to those remaining dementia free [11].

#### PD and PDD—apathy treatments

Moretti and colleagues conducted an RCT on the effect of rivastigmine on apathy and addressed apathy as the most constant NPS in PDD. Patients first received a 9.5 mg/24-h dose of transdermal rivastigmine for 3 months. Therefore, a maintenance dose of rivastigmine was administered for 12 months. The first evaluation reported that 77% of patients experienced apathy, with a serious impact on daily life and with severe relevance to caregivers. After 6 months, apathy decreased in prevalence (62%), but still severely affected daily living. After 12 months, the levels of apathy increased up to 69%, while other NPS (irritability, disinhibition, and euphoria) improved [16]. Camargo et al. did not evidence a correlation between disease duration or duration of levodopa therapy and depression or apathy. However, they indicated a correlation between motor changes and NPS. More specifically, depression scores were greater in patients with more severe motor impairment, but apathy scores did not correlate with worsening motor symptoms [15].

#### PD and PDD—Neurological correlates of apathy

No studies exploring neural correlates of apathy in PD or PDD as a primary outcome were found.

### Frontotemporal dementia

FTD is a group of neurodegenerative diseases characterized by four clinical variants distinguished by early and predominant symptoms: bvFTD, PPA, semantic variant PPA (svPPA), and nonfluent variant PPA (nfvPPA) [74]. bvFTD is the most common subtype and presents with a heterogeneous combination of socio-affective symptoms and executive deficits [75, 76]. In 2011, the consensus diagnostic criteria for bvFTD proposed six core features of the disease, namely, apathy, disinhibition, loss of empathy, change in eating behavior, stereotypical behavior, and executive dysfunction [75]. A summary of studies addressing apathy in FTD included in the review is shown in Table 3.

**Table 3:** Summary of studies addressing apathy in frontotemporal dementia included in the review

Author(s), year	Study design	Dementia	Participants, mean age, male/female (%)	Prevalence, %	Apathy evaluation	Mean apathy score (SD)	Task/other			Primary outcome		Results		
Alfano et al., 2021	Cross-sectional study	FTD PD	*N*: 53 20 apathy patients: 7 with primary diagnosis of FTD 64.6 (7.9) 13 with primary diagnosis of PD 67.0 (7.3) 20 non apathy patients: 7 with FTD 68.3 (7.2) 13 PD (67.7 (8.2)		AES		MRI fMRI			The study aims to investigate large-scale brain networks involved in apathy syndrome in patients with FTD and PD compared to a group of healthy controls (HC)		Results highlighted a significant hypoconnectivity (FC reduction) between apathetic patients (both FTD and PD) and HC detected between left planum polare and both right pre- or postcentral gyrus		
Fernàndez-Matarrubia et al., 2017	Cross-sectional study	bvFTD AD	*N*: 72 42 bvFTD 71.6 (8.3) 25/17 30 AD 12/30	76.2% 54.8%	LARS FBI-apathy NPI-apathy	LARS: bvFTD 8.29 (15.00) AD 4.52 (17.99) FBI-apathy: bvFTD 2.42 (1.12) AD 1.56 (1.42) NPI-apathy: bvFTD 9.43 (4.42) AD 5.29 (5.27)	MMSE ACE CDR FAQ FAB NPI HDR PET			The main purposes were to compare the clinical apathy profile from patients with bvFTD and AD and analyze the relationship between apathy and brain metabolism measured using positron emission tomography imaging with 18F fluorodeoxyglucose (FDG-PET)		These results support that apathy is a complex syndrome, with different clinical expressions across different pathological conditions. Therefore, subjects affected by bvFTD displayed greater impairment of emotional apathy and self-awareness in comparison with AD sample. Those differences in qualitative aspects of apathy seem to be associated with different functional and neuroanatomical substrates, as shown by FDG-PET imaging analysis		
Godefroy et al., 2021	Cohort study	bvFTD AD	*N*: 40 (couples) 40–85 years old divided into three groups: 1) 20 patient-caregiver dyads with patients diagnosed with bvFTD 2) 20 patient-caregiver dyads with patients diagnosed AD		AES		MADRS GSRs GSL HRV ESS			The main objective of this observational study was to define a behavioral signature of apathy using an ecological approach. called ECOCAPTURE@HOME which aims to validate a method based on new technologies for the remote monitoring of apathy in real life		The first step of this study proves that this protocol could be useful for the implementation of new treatments. In particular, by enabling the daily assessment of behavioral markers of apathy and associated dyad’s psychological health status, this system could be of great use to test and adapt therapeutic interventions accordingly in patients’ homes		
Kumfor et al., 2018	Cross-sectional study	bvFTD AD	*N*: 69 62.9 (7.9) 35/34 *N*: 53 62.1 (6.4) 28/25	84% 60%	NPI CBI-R		MRI FRS ACE DAD			The main purposes were to compare the clinical apathy profile from patients with bvFTD and AD and analyze the relationship between apathy and brain metabolism measured using positron emission tomography imaging with 18F fluorodeoxyglucose (FDG-PET)		The results of this study showed that both severity and nature of apathy are significantly different in AD and bvFTD. Although a relatively similar proportion of patients with AD and bvFTD showed apathy at the NPI score, in AD patients the apathy was less frequent and less severe than bvFTD patients		
,Lansdall et al., 2018	Cross-sectional study	FTD	*N*: 69 22 PSP 71.4 (7.4) 12/10 14 bvFTD 63.9 (7.4) 8/6 14 CBS 66.9 (8.0) 7/7 19 PPA 71.2 (7.5) 11/8		AES NPI (apathy subscore) MEI SHAPS	AES: PSP 39.7 (10.9); bvFTD 32.6 (10.2); CBS 35.2 (5.7); PPA 37.6 (6.3) NPI (Apathy Subscore): PSP 0.60 (0.50); bvFTD 0.71 (0.47); CBS 0.71 (0.47); PPA 0.42 (0.51) MEI: PSP 67.5 (30.4); bvFTD 97.3 (24.9); CBS 76.9 (25.6); PPA 86.7 (14.6) SHAPS: PSP 22.4 (4.7); bvFTD 23.5 (6.3); CBS 23.1 (5.7); PPA 20.6 (3.8)	MRI BAS BDI-II			The objective of this study was to identify the white matter correlates of apathy and impulsivity in the major syndromes associated with frontotemporal lobar degeneration		The results have evidenced three components that were associated with significant white matter tract abnormalities. Carerrated change in everyday skills, self-care, and motivation correlated with wide-spread changes in dorsal fronto-parietal and corticospinal tracts		
Pengo et al., 2022	Cohort study	byFTD PPA	N: 531 FTD 65.9 ± 8.3 273/258 354 bvFTD 118 avPPA 68 svPPA FTD-related pathogenic mutations were identified in 95 patients (*n*: 66 GRN mutations, *n*: 26 C9orf72 expansions, *n*: 3 MAPT mutation		FBI	FBI A All 12.2 ± 7.5 M 12.4 ± 7.5 F 12.0 ± 7.6 FBI B All 5.9 ± 5.8 M 6.8 ± 6.0 F 5.0 ± 5.4 FBI AB All 18.1 ± 11.7 M 19.1 ± 12.1 F 17.0 ± 11.3			MMSE TMT MRI PET NfL		The aim of this study was to estimate the role of sex in a single-center large cohort of FTD patients			The behavioral variant of FTD was more common in men, whereas primary progressive aphasia was overrepresented in women While global cognitive impairment was comparable, females had a more severe cognitive impairment On the other hand, men exhibited more personality/behavioral symptoms
Lansdall et al., 2019	Cross-sectional study	FTD	*N*: 124 35 PSP 72.2 (8.5) 19/16 29 CBS 68.9 (8.3) 13/16 33 PPA 71.5 (6.6) 15/18 27 bvFTD 63.5 (7.9) 15/12		AES MEI NPI CBI SHAPS				ACE BDI BAS BIS SST CRRT		The objective was to determine the influence of apathy, impulsivity, and behavioral change on survival in patients with frontotemporal dementia, progressive supranuclear palsy, and corticobasal syndrome			The current study provides evidence of distinct structural network changes in white matter associated with different neurobehavioral components of apathy and impulsivity across the diverse spectrum of syndromes and pathologies associated with frontotemporal lobar degeneration
Malpetti et al., 2020	Cross-sectional study	FTD	N: 304 304 presymptomatic mutation carriers (54 with mutation in MAPT, 142 in GRN, and 108 in C9orf72 44.5 (12.1) 187/117		CBI-R	CBI-R (apathy baseline) 0.3 (1.5)			MRI Digit Span (Backwards from the Wechsler Memory Scale-Revised) TMT B WAIS-R (Digit Symbol Substitution test)		In this study, they tested the hypothesis that apathy increases over time in presymptomatic carriers of FTD mutations are more severe in those closer to symptom onset			In this study, they found that apathy progresses significantly in presymptomatic carriers of mutations associated with FTD and that this progression of apathy over 2 years is associated with atrophy of the frontal lobe and cingulate gyrus at baseline
Musa Salech, 2022	Cross-sectional study	bvFTD	N: 27 patients with bvFTD in its early stage 69.1 (9.4) 17/10	88%	NPI-Q				ADL MMSE FAB FAS (and animal version of the COWAT) Digit Span Backward Task mini-SEA		The main purpose of this study was to examine and explore the association between the cognitive and neuropsychiatric features that might prompt functional impairment of basic, instrumental, and advanced ADL domains in patients with bvFTD			The results of this study showed the prominent and transverse effect of apathy in the loss of functionality throughout all the ADL domains. Apart from that, this is the first study that shows that the factors associated with loss of functionality differ according to the functional domain in patients with bvFTD in its early stage
O’Connor et al., 2017	Cohort study	bvFTD	*N*: 88 26 PSA 63.96 (7.93) 15/11 26 SA + D 60.00 (10.52 16/10 27 MA + D 62.30 (7.28) 17/10 9 PSD 62.67 (8.87) 6/3 Tot: 62.15 (8.68) 54/34		CBI-R	CBI-R 4subgroups: 64.641			MRI ACE-III DAD		The aim of this study was to identify distinct behavioral phenotypes of bvFTD and elucidate differences in functional, neuroimaging, and progression to residential care placement			This study reveals that different clinical behavioral phenotypes of bvFTD have differing profiles of functional decline and distinct patterns of associated cortical changes
Radakovic et a.l, 2020	Cohort study	bvFTD	*N*: 72 12 bvFTD 61.0 (11.9) 8/4 12 PPA 63.2 (6.7) 7/5 28 AD 62.5 (5.6) 16/12	83.3% 66.7% 57.1%	DAS AES	3 subscales: (executive apathy, emotional apathy, and initiation apathy) AES: bvFTD 55.3 (9.5) PPA 41.3 (12.1) AD 42.9 (9.3)			GDS-15 LIADL ECAS ACE		The aim of this study was to explore the apathy profile and awareness of apathy subtypes in bvFTD and PPA in comparison to AD and determine any relationships to cognitive functioning and activities of daily living			The results suggest that while patients with bvFTD displayed the highest levels of apathy over all subtypes, emotional apathy seems to be consistently characteristic in terms of bvFTD, when compared to AD, PPA, and controls
Valotassiou et al., 2020	Cross-sectional study	FTD AD	*N*: 144 75 FTD 65.8 (8.5) 27/48 66 AD 70.2 (8.0) 21/45		NPI	NPI: FTD 6.6 (4.2) AD 3.9 (4.0)		MMSE ACE-R CT MRI			The objective was to explore differences of apathy perfusion correlates between AD and FTD using perfusion SPECT		They found a considerable overlapping of apathy neural correlates between AD and FTD in bilateral frontal areas and anterior cingulate cortex. These findings suggest that the neurobiology of apathy in AD and FTD is complex and mediated by various brain regions, depending on the location of neurodegenerative pathology which affects different parts of apathy brain circuits	
Zhou et al., 2020	Cross-sectional study	bvFTD	*N*: 64 17 bvFTD (no apathy) 71.4 (2.2) 7/10 47 bvFTD (apathy) 72.2 (1.2) 26/21		NPI	NPI: bvFTD (no apathy) 20.35 (5.47) bvFTD (apathy) 38.11 (3.45)		MMSE MoCA HDR ADL SPECT			The aim of this study was to use the topological characteristic of CBF network to investigate the mechanism in bvFTD		In this study they investigated the topological characteristic of CBF network in bvFTD patients with and without apathy. They discovered that both of the bvFTD groups preserved global function and typical features of small worldness, but exhibited the loss of hubs mainly distributed in the PFC	
Min Chu et al., 2023	Cohort study	bvFTD	*N*:60 30 byFTD 62.60 ± 7.16 13/17 30 healthy control 63.6 ± 5.95 13/17		FBI-apathy	FBI-apathy byFTD 16.33 ± 5.18 Healthy control 0.17 ± 0.46		MMSE MRI DTI			The aim of this study was to investigate the clinical relevance of WM topological alterations in bvFTD		In this study the WM topological alterations in bvFTD were investigated. Compared to controls, bvFTD data showed that the right superior orbital frontal gyrus was associated with apathy and disinhibition	
Wong et al., 2022	Cross-sectional study	byFTD AD SD	*N*:91 33 byFTD 14 AD 8 SD 6 progressive nonfluent aphasia 3 logopenic progressive aphasia 27 healthy controls		DAS	DAS Emotional apathy Control 6 7.16 ± 4.01 LEA 9.94 ± 3.37 HEA 18.96 ± 3.01 DAS Executive apathy Control 6 3.68 (3.80) LEA 11.64 (5.14) HEA 16.89 (5.34) DAS Initiation apathy Control l6 7.20 (5.13) LEA 13.58 (4.83) HEA 18.00 (3.93)		ACE III DAS MRI			The aim of this study was to examine the link between emotional apathy and socioemotional processing, and their common neural correlates		This study shown a unique link between impaired social reward learning and emotional apathy in dementia and reveal a shared neurobiological basis. Greater understanding of these neurocognitive mechanisms of reward processing will help improve the identification of emotional apathy in dementia and inform the development of novel interventions to address these symptoms	

*AD* Alzheimer’s Disease, *ADL* Activities of Daily Living, *ACE* Addenbrooke’s Cognitive Examination, *AES* Apathy Evaluation Scale, *BAI* Beck Anxiety Inventory, *BAS* Behavioral Activations, *bFTD* behavioral Frontotemporal Dementia, *BIS* Barratt Impulsiveness Scale, *BDI* Beck’s Depression Inventory, *CBF* Cerebral Blood Flow, *CBI*-*R* Cambridge Behavioral Inventory-Revised, *CBS* Corticobasal Syndrome, *COWAT* Controlled Oral Word Association Test, *CDR* Clinical Dementia Rating scale, *CRRT* Cued Reinforcement Reaction Time task, *CT* Computed Tomography, *DAD* Disability Assessment for Dementia Scale, *DAS* Dimensional Apathy Scale, *DLB* Dementia with Lewy Bodies, *ECAS* Edinburgh Cognitive and Behavioral ALS Screen, *ESS* Epworth Sleepiness Scale, *FAB* Frontal Assessment Battery, *FAQ* Functional Activities Questionnaire, *fMRI* functional Magnetic Resonance Imaging, *FRS* Frontotemporal Dementia Rating Scale, *FTD* Frontotemporal Dementia, *GDS-15* Geriatric Depression Scale–Short Form, *GSL* Galvanic Skin Level, *GSRs* Galvanic Skin Responses, *HEA* High Emotional Apathy, *HC* Healthy Controls, *HDR* Hamilton Depression Rating Scale, *HRV* Heart Rate Variability, *LARS* The Lille Apathy Rating Scale, *LEA* Low Emotional Apathy, *LIADL* The Lawton Instrumental Activities of Daily Living, *MEI* Motivation and Energy Inventory, *MADRS* Montgomery-Åsberg Depression Rating Scale, *MMSE* Mini Mental State Examination, *MOCA* Montreal Cognitive Assessment, *MRI* magnetic resonance (structural), *mini-SEA* mini-Social cognition and Emotional Assessment, *NPI* neuropsychiatric inventory, *PDD* Parkinson’s Disease Dementia, *PET* Positron Emission Tomography, *PPA* Primary Progressive Aphasia, *PSP* progressive supranuclear palsy, *SD* Semantic Dementia, *SHAPS* Snaith-Hamilton Pleasure Scale, *SPECT* Single-photon emission computerized tomography, *SST* Stop Signal Task, *TMT B* Trail Making Test B

#### FTD—apathy prevalence

The prevalence of apathy in FTD was 54.8–88.0%. Apathy showed the highest reported frequency in FTD including bvFTD in ten studies [12, 21, 23, 76–82], while only two studies addressed PPA [76, 78]. In studies comparing FTD with AD, FTD patients had a higher prevalence of apathy [12, 21] and higher apathy scores [83, 84] than AD patients. Only one study highlighted a higher prevalence of apathy in patients with bvFTD in its early stage [82].

#### FTD—apathy assessment

Eight studies used NPI [21, 76, 78, 80, 82, 84–86], and five studies used AES [14, 23, 78, 81]. One study used LARS [12], three used CBI-R [21, 76, 77], two used the Motivation and Energy Inventory (MEI) [76, 78], three used the Snaith-Hamilton Pleasure Scale (SHAPS) [76, 78, 79], one used DAD [21], two used the Social and Emotional Assessment (Mini-SEA) [82, 87], one used AS [87], DAS was used in four studies [23, 79, 83, 88], one used the goal-directed behaviors (GDB) [81], and one used FBI [89]. Interestingly, one of the studies investigating bvFTD phenotypes related to WM changes found discordant scores on several apathy administration tests [78].

#### FTD—apathy treatments

None of the selected articles proposed pharmacological treatments for apathy. Godefroy et al. used a remote monitoring system named ECOCAPTURE@HOME to assess the evolution of apathy in individuals with dementia and its associated impact on their caregivers. They recruited 60 dyads of patients and caregivers (20 bvFTD, 20 AD, and 20 healthy control). Each dyad was monitored through a multisensory wearable bracelet and questionnaires on a smartphone application for 28 consecutive days. Building on the knowledge acquired through this first ECOCAPTURE@HOME study, they expected that the following phase of such a process will be to test a machine learning system, which could automatically estimate the behavioral markers of apathy and the associated caregiver’s perception of the dyad’s status using solely passive data from sensors [81].

#### FTD—neurological correlates of apathy

Included studies revealed that different clinical phenotypes of FTD have different profiles of functional decline and distinct patterns of associated cortical changes. Seven studies used MRI [14, 21, 77–79, 84, 85, 90]. One study used PET [12], and another one used SPECT [80]. In one study, 88 patients with bvFTD were included in a cluster analysis focusing on levels of disinhibition and apathy. Four phenotypic subgroups were identified in this study: primary severe apathy (*n* = 26), severe apathy and disinhibition (*n* = 26), mild apathy and disinhibition (*n* = 27), and primary severe disinhibition (*n* = 9). Results showed that apathy scores were associated with increased atrophy in the insula, inferior frontal, and anterior temporal cortex. Patients with severe apathy phenotypes were more functionally impaired with more extensive brain atrophy than those with mild apathy or primary severe disinhibition [77]. In another study, WM correlates of apathy and impulsivity were identified in the major syndromes associated with frontotemporal lobar degeneration, using diffusion-weighted imaging. The results highlighted three components associated with significant WM tract abnormalities: (1) care-rated change in daily skills, (2) changes in self-care, and (3) motivation correlated with widespread changes in dorsal frontoparietal and corticospinal tracts. In the neuropsychological tests of cognitive control, reflection impulsivity, and reward responsiveness were associated with focal changes in the right frontal lobe and supplementary motor area [78]. Another study showed that bvFTD subjects had greater impairment of emotional apathy and self-awareness than the AD sample. Such differences in the qualitative aspects of apathy appear to be associated with different functional and neuroanatomical substrates, as shown by FDG-PET imaging analysis of left lateral prefrontal, medial frontal/anterior cingulate, lateral insular cortex, and lateral OFC in bvFTD and right ACC [12]. Furthermore, neuroimaging results showed that among dementia syndromes (bFTD and AD), greater affective apathy was associated with reduced GM intensity in the ventral PFC and cognitive apathy with dorsal PFC. The presence of behavioral apathy was associated with the degradation of subcortical regions, including the caudate region of the basal ganglia, as well as the premotor cortex and cerebellum [21]. Moreover, they found that apathy progressed significantly in pre-symptomatic carriers of FTD-associated mutations and those individual differences in apathy at baseline predicted the severity of progressive deterioration in digit symbol test performance over time. In pre-symptomatic carriers, progression of apathy over 2 years was associated with baseline frontal lobe and cingulate gyrus atrophy. In contrast, subclinical cognitive impairments did not predict the worsening of apathy [90]. Another study investigated the topographic feature of the rCBF network in bvFTD patients with and without apathy. They found that both bvFTD groups retained the global function and characteristics typical of small mundanity but showed the loss of hubs distributed mainly in the PFC. Compared with the bvFTD no-apathy group, the bvFTD apathy group showed further loss of hub in the frontostriatal circuit, but recruited hubs in the angular gyrus, precuneus, and PCC. Overall, their results support previous findings from other neuroimaging modalities, in which apathy was related to frontostriatal circuit disruption in bvFTD [80]. Concerning cognitive apathy, an MRI study demonstrated that the presence of a deficit in planning ability is a predictor for a worsening in cognitive decline in both bvFTD and AD and it shares similar pathways with apathy. More precisely, alterations in the lateral and medial prefrontal and lateral temporal cortices as well as subcortical regions including the hippocampus, striatum, and thalamus were detected in both patients with apathy affected by AD and bvFTD [88]. Finally, O’Connor et al. evaluated the importance of considering anhedonia as a primary presenting feature of bvFTD and semantic dementia, with neural drivers distinct from those of apathy or depression. Overall, the results underscore the importance of apathy in functional impairment, highlighting the role of the right temporal region in disinhibition [77]. Lansdall and colleagues also suggested that apathy and impulsivity are correlated with WM tract abnormalities in the major syndromes associated with frontotemporal lobar degeneration, while daily skills, self-care, and motivation were probably related to diffuse changes in the frontoparietal and dorsal corticospinal tracts [78].

## Discussion

### Prevalence of apathy in different forms of dementia

Apathy was shown to have broad prevalence intervals in all types of dementia (26–82% for AD, 28.6–91.7 for VaD, 29–97.5% for PDD, and 54.8–88.0 for FTD). One possible explanation for the ambiguous prevalence is the use of multiple tools to quantify apathy and dementia severity. Most studies assessed the severity of dementia using MMSE, which may be not sufficiently accurate in moderate-to-severe dementia [91]. Nonetheless, while apathy appears to be more often connected with later stages of dementia, previous studies demonstrated that it could be prodromal for executive functions deterioration and also associated to an increased risk of developing cognitive decline in community-dwelling people [92]. Moreover, although apathy underlies similar symptoms to other disorders, such as depression [93], agreement on a standardized diagnostic system has not been established yet. Finally, except for a few research [14–16], all the included studies did not have apathy as their primary outcome. No studies investigating predictive risk factors for dementia primarily investigated the role of apathy, as opposed to depression or to other behavioral disorders. This has made it even more complex to outline a clear profile of apathy in relation to patients with different forms of dementia, although apathy is extremely related to these conditions.

### Apathy assessment in different forms of dementia

The use of multiple diagnostic techniques for assessing apathy may explain the differences in outcomes. Several studies employed generic scales, such as the NPI, which might lead to inaccurate identification. Apathy is frequently underestimated and misdiagnosed as depression [4]. Due to the necessity for specialist training, apathy-specific measures like the AES are seldom used in clinical practice. Apathy has been discovered as a predictive biomarker for the likelihood of acquiring major diseases such as PD, cognitive decline, and severe dementia [94, 95]. An early diagnosis of apathy could help clinicians to detect dementia in earlier stages, as well as prevent frailty [96] or a misdiagnosis of depression. For these reasons, a more specific test appears to be important in assessing therapy response across multiple domains [97]. Given the prevalence of several phenotypes of apathy, the NPI appears to be a deficient scale in clinical practice for diagnosing apathy, whereas AES [98], DAS, or LARS appeared more appropriate to investigate the domains of apathy. More specifically, a systematic review reported that DAIR and AES clinician versions for AD, and LARS for PD, have the highest methodological quality and psychometric properties among the other validated apathy scales [99]. Correct diagnosis of apathy has substantial consequences for caregiver quality of life, [16] and it is likely that caregiver distress has important implications on the perception of the apathetic behavior of their assisted. Thus, employing a unique neuropsychological instrument and distinguishing scales administered by caregivers from those administered by clinicians may be relevant in future research to show possible variations in apathy identification and quantification due to the type of clinical scale.

### Apathy treatments in different forms of dementia

Despite a recent increased research interest in apathy in dementia, the number of RCT for pharmacological treatments is poor. Methylphenidate produced the most meaningful findings for apathy in AD, as it improved different apathy domains. However, included studies reported discordant results on cognitive performance and caregiver distress [45, 51]. However, in these circumstances, the inclusion of non-apathy-specific testing may have a detrimental impact on the study’s results. rTMS paired with cognitive training was found to significantly enhance apathy as well as cognitive functioning among non-pharmacological therapies in AD [40]. However, as noted by a systematic review by Theleritis and colleagues, apathetic patients with dementia may benefit from personalized treatments combining both pharmacological and non-pharmacological interventions, including lifestyle changes and environmental adjustments [100].

### Neurological correlates of apathy in different forms of dementia

The neurological basis of apathy is thought to be a disruption in networks controlling goal-directed behavior. Although the PFC is one of the key regions engaged in these tasks [4], changes in other brain regions might result in distinct phenotypes of apathy [101]. Recently, a new categorization of apathy was developed based on clinical findings of individuals with damage to PFC and basal ganglia [102]. Emotional apathy seems to be due to lesions in the connection between ventral-striatum fibers and orbitomedial PFC; cognitive apathy seems to involve neural connections between lateral PFC and dorsal caudate nuclei; auto-activation apathy seems to be due caused by bilateral lesions in limbic and cognitive territories in basal ganglia (internal globus pallidus and bilateral paramedian thalamus) and dorso-medial part of PFC. The finding of the neurological mechanisms underlying these illnesses is intimately connected to the function of apathy as a risk factor for neurodegenerative diseases. Neuroimaging studies in AD identified brain areas primarily related with apathy; nevertheless, even in this situation, the multiple neuropsychological tools employed to quantify apathy degree resulted in diverse and sometimes contradictory results. Indeed, Huey et al. using NPI found a greater degree of atrophy in ventromedial and ventrolateral PFC, posterior cingulate cortex, and the superior temporal sulcus [24]. Instead, Aguera-Ortiz L. et al., using the Apathy in Dementia Nursing Home Version Scale, addressed bilateral damage of the corpus callosum and internal capsule [37]. Similarly, to evaluate brain atrophy related to different apathy domains, Wei et al. applied the DSA [22], while Kumfor et al. used NPI, CBI-R, and DAD [9]. In the first study, they found GM atrophy along the brain regions implied in emotional apathy, executive apathy, and initiation apathy [22]. Conversely, in the second study, GM atrophy was detected in affective apathy, behavioral apathy, and cognitive apathy. These results demonstrated that the use of different neuropsychological tests may lead to heterogeneous and misleading data; therefore, an apathy-specific tool is recommended. Studies investigating the neuroanatomical substrate of apathy in AD and VaD supported the disruption of pathways connecting brain regions implicated in effort-based decision-making (disconnection theory) as the neurological substrate of apathy [13, 65–67]. In comparison to AD patients, FTD patients had a more widespread emotional deficiency, which was related with decreased self-awareness. According to an FDG-PET investigation, changes in qualitative characteristics of apathy appear to be related with several functional and neuroanatomical substrates [12]. Another neuroimaging study found that in both AD and bvFTD, affective apathy was associated with reduced GM intensity in the ventral PFC, cognitive apathy with dorsal PFC, and behavioral apathy with degradation of subcortical regions, premotor cortex, and cerebellum WM correlates of apathy and impulsivity were related with frontotemporal lobar degeneration in FTD using DTI. These WM anomalies, which were linked to alterations in daily skills, self-care, and motivation, were shown to be associated to changes in the dorsal frontoparietal and corticospinal circuits. The neuropsychological tests, on the other hand, yielded different results, linking these behavioral changes to alterations in the right frontal lobe and pre-supplementary motor area [78]. In conclusion, in both AD and VaD, apathy increases as dementia and WM atrophy progress [64, 103]. In FTD, the role of the right temporal region in disinhibition has been consistently highlighted, together with the correlation of apathy with WM tract abnormalities [77]. No data on neuroimaging correlates have been reported for PDD.

## Conclusions

Despite its high frequency in neurodegenerative disorders, apathy is a neurobehavioral condition that is too often misdiagnosed. This review highlights that the appropriate diagnostic methods are still not employed in both clinical practice and research, as well as a paucity of studies that examine apathy as a primary outcome. Inadequate assessment leads to a lack of evidence on appropriate treatments for this disease. Possible therapeutic strategies to modulate this condition could be neurostimulation or cognitive training. However, agreement on the various assessment measures should be reached in order to test and reduce discrepancies between clinical studies. As far as we know, this is the first review that reports the literature on apathy based on the most common dementias. As the main limitation, the present study reflects the findings of a narrative review.

## Data Availability

No new data were created or analysed in this study. Data sharing is not applicable to this article.
